# MicroRNAs Regulate Vascular Medial Calcification

**DOI:** 10.3390/cells3040963

**Published:** 2014-10-14

**Authors:** Jane A. Leopold

**Affiliations:** Division of Cardiovascular Medicine, Brigham and Women’s Hospital and Harvard Medical School, 77 Avenue Louis Pasteur, NRB0630K, Boston, MA 02115, USA; E-Mail: jleopold@partners.org; Tel.: +1-617-525-4846; Fax: +1-617-525-4830

**Keywords:** microRNAs, matrix vesicles, osteoclasts, vascular smooth muscle cells, vascular calcification

## Abstract

Vascular calcification is highly prevalent in patients with coronary artery disease and, when present, is associated with major adverse cardiovascular events, including an increased risk of cardiovascular mortality. The pathogenesis of vascular calcification is complex and is now recognized to recapitulate skeletal bone formation. Vascular smooth muscle cells (SMC) play an integral role in this process by undergoing transdifferentiation to osteoblast-like cells, elaborating calcifying matrix vesicles and secreting factors that diminish the activity of osteoclast-like cells with mineral resorbing capacity. Recent advances have identified microRNAs (miRs) as key regulators of this process by directing the complex genetic reprogramming of SMCs and the functional responses of other relevant cell types relevant for vascular calcification. This review will detail SMC and bone biology as it relates to vascular calcification and relate what is known to date regarding the regulatory role of miRs in SMC-mediated vascular calcification.

## 1. Introduction

Vascular calcification is a highly prevalent phenotype that is identified frequently in patients with atherosclerosis, diabetes mellitus and chronic kidney disease (CKD). Community-based studies have also demonstrated that vascular calcification occurs as a consequence of aging with detectable coronary artery calcification present in ≥90% of men and ≥67% of women who are age 70 years or older [1,2]. In other population-based studies, the finding of ectopic vascular calcification has been associated with major adverse cardiovascular events. One meta-analysis that included 218,080 patients that were followed for ~10 years found that the presence of vascular calcification was associated with an odds ratio for cardiovascular mortality of 3.94 (95% CI: 2.39–6.50) [3].

Vascular (coronary artery) calcification is a well-accepted marker of increased cardiovascular risk, and compared to individuals with a coronary artery calcium score (CAC) = 0, those with a score >300 have a 10-fold increased risk for any cardiovascular event [4,5]. Moreover, CAC scores have been shown to be better predictors of risk than other markers, such as C-reactive protein, family history and the ankle-brachial index [6]. The addition of the CAC score to risk prediction models improves the classification of patients at risk for adverse cardiovascular events, and pooled data revealed that the annual rate of cardiac death or myocardial infarction was 0.4%, 1.3% or 2.4%, respectively, with each increasing tertile of CAC (<100, 100–399 and >400) [7]. These data also revealed that individuals with intermediate risk scores and high CAC scores (>400) had a coronary heart disease equivalent risk with up to a 20% event rate over 10 years [8]. Studies have evaluated the added information gained by nuclear stress testing with myocardial perfusion imaging. In one study of 462 patients that underwent coronary CT scan and single-photon emission computed tomography (SPECT)-myocardial perfusion imaging (MPI), those individuals that were found to have matched areas of calcification and perfusion defects had worse survival over a mean of 34.5 ± 13.0 months follow-up [9]. Coronary artery calcification was also shown to provide stepwise incremental information regarding future adverse events in patients with or without ischemia detected by positron emission tomography perfusion imaging [10]. Currently, American Heart Association guidelines suggest that cardiac CT to identify vascular calcification may be beneficial for individuals with an intermediate Framingham Risk Score [8]. The aforementioned studies all support the concept that CAC is linked to increased cardiovascular risk; however, to date, there are no data demonstrating that CAC is causal for adverse cardiovascular events.

Vascular calcification is typically localized to neointimal plaques that encroach upon the vessel lumen in atherosclerotic vessels, where it is present in a punctate or patchy pattern [11]. Vascular calcification also occurs in the medial layer of the vessel, which is known as Monckeberg’s medial sclerosis, where mineralization can be widespread, with a predilection for the vascular smooth muscle cells (SMC), the elastic lamellae and the extracellular matrix [12].

The pathogenesis of vascular calcification is a complex multifactorial process that has been attributed to inflammation, disorders of metabolism and genetic causes. Vascular medial calcification, in particular, has been shown to occur in diseases, such as diabetes mellitus and chronic kidney disease, which are associated with abnormal metabolic profiles. Calcification of the media has also been linked to a number of monogenetic disorders (e.g., polymorphisms in the genes *MGP*, *OPG*, *ENPP1* and *NTE5*) that mainly affect calcium-phosphate homeostasis [13,14]. The complexity of vascular medial calcification is underscored further by the fact that this is likely a multicellular process with the involvement of osteoprogenitor cells, inflammatory cells and vascular cells that together recapitulate the cellular processes related to skeletal bone formation and remodeling [13,14,15,16].

One component of the vascular calcification process involves the reprogramming and transdifferentiation of SMC to osteoblast-like cells. These osteoblast-like SMCs generate and release calcifying matrix vesicles that are another essential factor involved in vascular calcification [17]. Concomitant loss of circulating inhibitors of vascular calcification; increased cellular stress, such as oxidant stress, endoplasmic reticulum stress or DNA damage response signaling, apoptosis and cell death and disorders of calcium-phosphate homeostasis all contribute to creating the milieu that favors mineral deposition in the vessel wall and frank calcification.

As the process of vascular calcification is tightly regulated and involves genetic reprogramming of SMC and dynamic regulation of pro-calcifying peptides and inhibitors, it is not surprising that there is accumulating evidence to support an integral role for microRNAs (miRs) in this process. Emerging data has established that miRs regulate several key checkpoints in the cellular processes involved in vascular calcification. This review will briefly summarize the cellular and molecular mechanisms underlying vascular calcification and present evidence supporting the role of miRs in regulating integral processes for vessel wall mineralization.

## 2. Mechanisms of Bone Formation

Vascular calcification is now recognized to occur as a result of the same processes involved in bone formation. Bone forming osteoblasts are derived from mesenchymal cells that progressively differentiate into proliferating preosteoblasts, osteoblasts capable of synthesizing bone matrix, and that eventually turn into osteocytes that are resident in mature bone. Runx2 is a master osteoblast transcription factor and is recognized as the earliest osteoblastic marker [18]. Runx2 regulates the expression of osteocalcin, sclerostin, dentin matrix protein 1, vascular endothelial growth factor and the receptor activator of nuclear factor-kappaβ ligand (RANKL) [19]. The transcription factor, osterix, which is downstream from Runx2, is necessary for osteoblast differentiation [20]. Osterix regulates the expression of the extracellular matrix protein, bone sialoprotein, as well as the proteoglycans, osteomodulin, asporin and osteoglycin [21]. Bone morphogenetic proteins (BMPs), angiogenic factors and growth factors also regulate osteoblast differentiation, in part by activation of Wnt signaling [15,22]. Wnt proteins promote osteoblast differentiation by binding to the low-density lipoprotein receptor-related protein-5 or -6 and one of the frizzled molecules to stabilize β-catenin and regulate transcription factor expression [22,23]. Canonical Wnt signaling favors osteoblast differentiation from mesenchymal cells at the expense of adipogenesis and is regulated by Runx2 and osterix [24,25].

Fully mature osteoblasts express type 1 collagen and alkaline phosphatase [26]. These mature cells also produce osteocalcin, osteopontin and osteonectin, which regulate matrix mineralization, as well as RANKL, which is necessary for osteoclast differentiation [27]. At the end of their lifespan, osteoblasts transform into osteocytes. Osteocytes are embedded into the mineralized bone matrix and express fibroblast growth factor-23 and sclerostin, which control phosphate metabolism and bone formation [27]. Another key feature of bone formation and remodeling involves osteoclasts. Osteoclast differentiation is regulated through the RANKL/RANK/osteoprotegerin signaling pathway. This pathway links bone forming osteoblasts, which synthesize RANKL, to osteoclast differentiation that occurs after RANKL binds to the membrane receptor RANK on osteoclast precursors [27]. Osteoclast differentiation by RANKL is inhibited by osteoprotegerin, which is a decoy receptor that binds RANKL to limit RANK activation and osteoclast differentiation [27]. Osteoprotegerin is also produced by osteoblasts [28].

During normal bone remodeling, bone resorption occurs concomitant with bone matrix elaboration and is matched to maintain bone density. This coupling of the two processes is suggested to occur as a result of growth factors and cytokines liberated during bone resorption. It also appears to involve signaling through sphingosine-1-phosphate, the transmembrane protein, ephrinB2, that is expressed on osteoblasts and the ephrin receptor B4 on osteoclasts [29]. Ephrin signaling by cell-cell contact stimulates osteoblast differentiation and represses osteoclast differentiation [29]. Conversely, sphingosine-1-phosphate, which is secreted by osteoclasts, leads to recruitment and differentiation of osteoblast progenitors. This shuts down bone resorption and initiates bone matrix elaboration [30].

A number of factors have been shown to be important for bone remodeling, including the BMPs, transcription factors Runx2 and osterix and Wnt signaling [31]. Other important factors include fibroblast growth factor-23 and klotho. Fibroblast growth factor-23 mediates renal phosphate excretion by inhibiting the sodium-phosphate transporters, NPT2a and NPT2c, to prevent reabsorption. Klotho was identified as a fibroblast growth factor-23 co-receptor that is required for these actions [32]. Emerging evidence indicates that there is an additional level of regulation that is mediated by small non-coding single-stranded RNAs or microRNAs (miRs) [33]. These ~22 nucleotide small RNAs are post-transcriptional regulators of gene expression that in disease states, such as vascular calcification, may regulate clusters of genes linked to disease pathogenesis.

## 3. miRs That Regulate SMC Transdifferentiation

**Figure 1:**
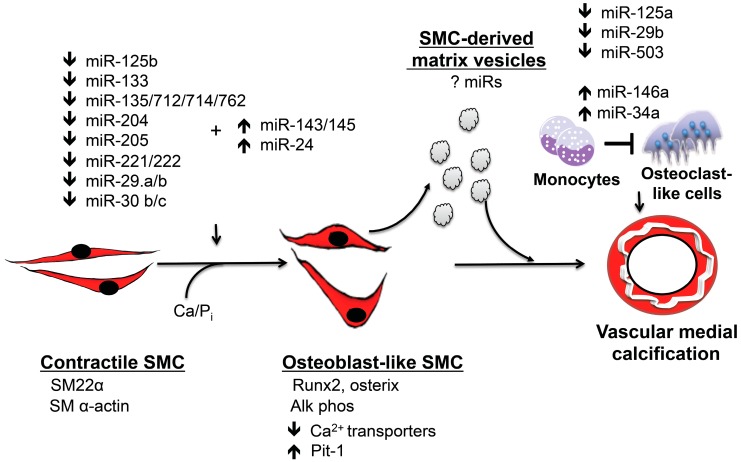
miRs regulate vascular medial calcification. miRs play an integral role in regulating vascular medial calcification by initiating vascular smooth muscle transition to calcifying vascular cells that express the master osteoblast transcription factors, Runx2 and osterix. These osteoblast-like cells also express alkaline phosphatase and elaborate bone matrix. Smooth muscle cell transdifferentiation occurs concomitant with the downregulation of smooth muscle cell contractile proteins. Calcifying smooth muscle cells generate matrix vesicles that contribute to vascular medial calcification by serving as a nucleation site for calcium phosphate. These matrix vesicles may also carry miRs that regulate phenotype in other cells. miRs also inhibit osteoclastogenesis and likely limit the bone resorptive capacity of osteoclast-like cells in the vessel wall to promote vascular calcification. This system has significant redundancy, and several miRs target the same protein or signaling pathway.

The transdifferentiation of SMCs to osteoblast-like cells that elaborate bone matrix is a recognized contributor to vascular calcification [14,34]. These calcifying SMCs have enabled a genetic program that allows them to resemble osteoblasts and utilize the same bone forming mechanisms for *in situ* calcification in vessels [34]. miRs play an integral role in SMC transdifferentiation by regulating Runx2 and osterix expression in conjunction with other pertinent signaling pathways (Figure 1).

Several studies have identified miRs that that are associated with calcification of SMCs *in vitro*, although these studies differ in the origin or vascular bed of SMCs studied, the pro-calcification conditions and the timing of analysis. In addition, many studies used a reductionist approach and examined only one miR and one target. Nonetheless, the totality of the studies indicates that there is redundancy in the system, with many miRs implicated in vascular calcification having the same target. For instance, miR-125b was the first miR to be associated with human coronary artery SMC calcification. After 21 days in osteogenic medium, miR-125b was decreased in these cells compared to controls. The transcription factor, osterix, was identified as a miR-125b target, and inhibition of miR-125 was associated with increased Runx2 and osterix expression, as well as increased alkaline phosphatase activity and SMC calcification. *In vivo*, miR-125b was downregulated in calcified aortas from 30-week-old apoE knockout mice. Interestingly, this study also reported that dicer and Drosha mRNA were downregulated in SMC under procalcifying conditions; however, protein levels were not examined, and it is not known if diminished expression of these processing enzymes had a global effect on other miRs [35]. Human aortic SMC, exposed to high phosphate conditions, demonstrated downregulation of miR-205 expression as early as three days after the initial exposure and by ~50% by 10 days. This occurred prior to the increased expression of its targets, Smad1 and Runx2, and cell mineralization [36].

Other studies found that miRs that target Runx2, including miR-133 and miR-204, are downregulated in murine aorta SMCs, leading to calcification *in vitro* [37,38]. miR-204, which is encoded in the transient receptor potential cation channel subfamily M member 3 gene, was also shown to regulate *in vivo* calcification in a vitamin D3 (500,000 IU/kg SQ daily day 1–3) murine model of vascular calcification. In this model, aorta calcification was associated with an increase in vascular Runx2 expression. Mice treated with agomiR-204 (80 mg/kg) to increase miR-204 expression had significantly less calcification and decreased aorta Runx2 expression and no effect on bone density, thereby confirming that manipulating miR expression levels may have therapeutic effects for vascular calcification [38].

Members of the BMP superfamily are known regulators of calcification, and BMP2 and BMP4 are recognized as osteogenic differentiation factors that have been identified in calcified atherosclerotic vessels [39]. BMPs activate calcification signaling via a receptor complex (type I and type II receptors) that stimulates Smad 1/5/8 signaling to initiate transcription of Runx2 [15]. BMP2 stimulates SMC calcification of human coronary artery smooth muscle cells by downregulating the expression of miR-30b and miR-30c. These miRs were shown to bind to the 3’-untranslated region of Runx2 to regulate its expression. The expression of miR-30b was found to be downregulated in calcified human coronary arteries, as well as in calcified stenotic human aortic valves [40,41]. These findings are also supported by data from a miR program analysis to determine an miR signature for BMP-2-induced osteogenesis in C2C12 mesenchymal cells. This study identified 22 downregulated miRs that included miR-133, miR-30 family members and miR-135 that targeted Runx2 and Smads [42].

It is also known that the wingless-type MMTV integration site family member (Wnt) is required for osteoblast function and is involved in SMC transdifferentiation [15]. Wnt/β-catenin signaling is activated in calcifying SMCs and is involved in a regulatory circuit that includes miR-29 [16,43]. Canonical Wnt signaling was shown to induce miR-29 expression. miR-29, in turn, negatively regulates the Wnt inhibitors, Dickkopf, secreted frizzled related protein 2, Kremen and osteonectin, to potentiate the calcification phenotype [43,44]. This has been shown in calcifying rat SMCs with decreased miR-29 a/b expression and confirmed in calcified aortas from mice and human radial arteries harvested from patients with CKD [45]. miR-29 a/b also targets the extracellular matrix modulating protein, disintegrin, and metalloproteinase and thrombospondin motifs-7 (ADAMTS-7). Transfecting cells with miR-29 mimics inhibited and miR antagomiRs enhanced SMC calcification under pro-calcific conditions by modulating ADAMTS-7, BMP-2, p-Smad 1/5/8 and Runx2 protein expression [45]. miR-29 was also associated with extracellular matrix remodeling that reduces the integrity of collagen and elastin. Therefore, downregulation of miR-29 may also promote vascular hypertrophy and fibrosis that facilitates mineralization [46].

## 4. miRs That Regulate Calcium and Phosphate Homeostasis

Disruptions to calcium and phosphate homeostasis also contribute to SMC transdifferentiation and vascular calcification [14]. Studies performed using klotho mutant mice (*kl/kl*) were analyzed to determine the role of miRs in regulating calcium and phosphate handling proteins important for vascular calcification. These mice develop precocious vascular calcification with elevated phosphate levels and increased vascular Runx2 expression [47]. Using an miR microarray to assess miR expression in the aortic media of three-week-old mice, 17 miRs were increased and three miRs were decreased (miR-1, miR-93 and miR-302b) in *kl/kl* mice, as compared to wild-type controls. The levels of miR-135a, miR-762, miR-714 and miR-712 were higher in calcified aortas of *kl/kl* mice as compared to wild-type mice, and this expression pattern was confirmed in SMC treated with calcium and inorganic phosphate. Bioinformatics revealed that these miRs target the calcium efflux proteins, sodium/calcium exchanger NCX1, plasma membrane Ca^2+^-ATPase-1 and sodium/calcium exchanger NCX4. Downregulation of the calcium transporters resulted in an increase in intracellular calcium concentrations, which has been implicated in SMC calcification. Interestingly, downregulation or upregulation of individual miRs from this group of four had no effect on SMC calcification, but inhibiting all four at the same time decreased calcium content by 30%. This led to the conclusion that the miRs are specifically involved in calcification and likely function as a cluster; however, this was not proven, and the significance of the other miRs identified in the expression array was not examined [48].

Studies have also identified miR-223 as an important regulator of vascular calcification in vascular SMCs exposed to high levels of inorganic phosphate. miR-223 is expressed in osteoclast precursors, is a known mediator of osteoclast phenotype and has been shown to limit RANKL-induced osteoclast differentiation. *In vitro*, miR-223 was upregulated in SMCs under pro-calcific conditions and found to promote SMC proliferation and migration, in part by decreasing the expression of its targets, Mef2 and RhoB [49,50,51]. Interestingly, miR-223 was also increased in aortas isolated from murine models of chronic kidney disease that are subject to vascular calcification. Here, upregulation of miR-223 was associated with downregulation of the transcription factor NFIA and the glucose transporter, GLUT-4, at later stages of disease [50,51].

To identify other miRs relevant miRs for SMC calcification, murine aorta SMCs grown in calcification medium for nine days were studied using miR microarrays. This identified more than 100 differentially-expressed miRs. Of these, several miRs (miR-221, -222, 24-2, 27a and 31) were confirmed to be downregulated *in vitro*. Further investigation revealed that miR-221 and miR-222 were found to act synergistically to induce calcification. Interestingly, these miRs do not target Runx2, but instead regulated the expression of ectonucleotide pyrophosphatase/phosphodiesterase 1 and Pit1 to influence phosphate metabolism [52]. To date, there are no comprehensive studies using miR microarrays to document changing miR profiles over time as SMCs transdifferentiate and mineralize. Studies also identified miR-9 as an important regulator of phosphate metabolism. miR-9 functions as a negative posttranscriptional regulator and downregulates expression of ectonucleotide pyrophosphatase/phosphodiesterase 1, Pit1 and alkaline phosphatase [53].

## 5. miRs That Downregulate the SMC Contractile Phenotype

In concert with SMC transdifferentiation and the acquisition of an osteoblast-like phenotype, calcifying SMCs exhibit downregulation of SMC contractile proteins, such as smooth muscle α-actin and smooth muscle myosin heavy chain [14,54]. This phenotype shift involves the miR-143/145 cluster that regulates the SMC differentiated phenotype, in part by targeting the transcription factor, myocardin [42]. Human SMCs exposed to high levels of inorganic phosphate that initiate SMC transdifferentiation also exhibit decreased miR-143 and 145 expression. Downregulation of miR-145 is believed to occur through activation of phosphatidyl inositol-3 kinase/Akt/p53 signaling, leading to impaired conversion of pri-miR-145 to pre-miR-145 [55]. Although the decrease in miR-143/145 expression is not involved directly in the pathogenesis of vascular calcification, there is an indirect relationship, as miR-145 targets Krüppel-like factor-4, which mediates high phosphate-induced transition of SMCs to osteogenic cells [56]. Other factors known to regulate miR-143/145 expression include brg1-containing SWI/SNF complexes that are required for myocardin to induce miR-143/145 and Notch/Jagged-1 signaling, which increases miR-143/145 in parallel with the serum response factor-myocardin complex that binds to CArG sequences to increase miR-143/145 transcription [57,58]. Therefore, the decreased expression or activity of any of these factors that upregulate miR-143/145 would promote the loss of SMC contractile proteins. This is in agreement with the finding that circulating miR-145 levels are reduced in patients with atherosclerotic coronary artery disease, a disease that is often associated with vascular calcification [49]. miR-24 also regulates SMC contractile marker gene expression. Using high-throughput screening and miRNA libraries, miR-24 emerged as a master regulator of the SMC phenotype. Increased expression of miR-24 was shown to target heme oxygenase-1 and decrease cellular stress resistance, leading to the activation of apoptosis, autophagy and the loss of contractile marker genes [59].

## 6. Matrix Vesicles and Circulating miRs

Transdifferentiated or calcifying SMCs generate extracellular matrix vesicles that serve as mineral nucleation sites and have been implicated as an important component of the vascular calcification process. These extracellular vesicles are released from SMC in response to elevated intracellular calcium levels. The increase in cytosolic calcium initiates translocation of annexin 6 to the plasma membrane, which, in turn, signals for the release of the matrix vesicles. These mineralization-competent vesicles contain noncrystalline calcium and phosphate, as well as other proteins. The initial calcium and phosphate ions are packaged as a Ca^2+^-P_i_-phosphatidylserine complex that serves as a core to nucleate hydroxyapatite (reviewed in [60]). Prior studies have demonstrated that SMC-derived matrix vesicles contain proteins. Mass spectrometry analysis of vesicles has identified at least 79 proteins that are contained within SMC-derived matrix vesicles. The vesicle proteins are related to processes involved in vascular calcification and include proteins that regulate calcification, calcium channels and extracellular matrix, matrix and cytoskeletal proteins, oxidant stress-related proteins, as well as other serum proteins [61]. Matrix vesicles also contain the calcium-dependent enzyme, transglutaminase 2, that crosslinks extracellular matrix and activates matrix metalloproteinase-2 to remodel the extracellular matrix and provide sites for mineral deposition [61,62].

There is accumulating evidence that matrix vesicles also contain miRs that may importantly regulate the phenotype of remote cells or tissues. The SUMOylated protein, heterogeneous nuclear ribonucleoprotein A2B1 (hnRNPA2B1), has been shown to bind miRs that contain the motifs GGAG and UGCA in the 3’ half of the miR sequence and mark these miRs for packaging in matrix vesicles [63]. In fact, several miRs are preferentially packaged in vesicles. RNA-seq analysis identified several miRs synthesized by porcine adipose tissue-derived mesenchymal stem cells, including miR148a, miR532-5p, miR378 and let-7f, which target genes relevant for apoptosis, angiogenesis, cellular transport and proteolysis, that were enriched in matrix vesicles compared to cells [64]. Other studies performed using preosteoblast cells identified levels of miR-122-5p, miR-451a, miR-183-5p, miR-144-3p and miR-142-5p as being ≥10-fold higher in matrix vesicles compared to cells [65]. This observation was supported by the finding that there are miRs that are commonly packaged in matrix vesicles across several different cell types. Analysis of the matrix vesicles and cellular miR content of HEK293T, human microvascular endothelial cells and primary outgrowth endothelial progenitor cells revealed that miR-451, miR-150, miR-720, miR-125a, miR-320a and miR-222 were included in the vesicles, while miR-10a, miR-26, miR-21, miR-20a, miR-376 and miR-216 were retained by the cells in roughly the same proportions in all three cell types [66].

It is also recognized that there is a population of circulating miRs in human plasma that are stable and independent of matrix vesicles. Thus, there are two populations of miRs in the systemic circulation, one that is enclosed in matrix vesicles and one that complexes with Argonaute2 (Ago2), a key component of the RNA-induced silencing complex. miRNA profiling arrays detected 88 plasma and 66 serum miRs. Of these, ~15% of the detected circulating miRs were found in eluted fractions consistent with matrix vesicles and 66% of plasma and 68% of serum miRs were enriched in fractions with Ago2. These findings were validated with select miRs, and similar to other studies, let-7a, miR-142-3p and miR-150 were identified in matrix vesicles; and miR-122 and miR-150 circulate with Ago2 complexes [67].

The functional consequences of circulating miRs for the vasculature have been demonstrated and provide novel insight into the regulatory function of one cell type over another. For example, endothelial matrix vesicles that were generated by cells exposed to shear stress were found to be enriched with miR-143 and miR-145. When co-cultured with SMCs, the endothelial-derived vesicles containing the miRs were shown to mediate the expression of miR-143/145 target proteins and, thereby, phenotype, in SMCs [68]. Others found that shear stress-stimulated endothelial cells secreted miR-126 that complexed with Ago2 to decrease forkhead box O3, insulin receptor substrate 1 and B-cell lymphoma 2 mRNA in the SMCs [69].

These functional interactions become important, as there are a few reports of associations between circulating miRs and vascular calcification in patients with chronic kidney disease (CKD) or coronary artery disease. In 90 patients with CKD Stage 3-4, circulating levels of miR-125b, miR-145 and miR-155, which target Runx2, the angiotensin type 1 receptor and myocardin, were decreased compared to levels measured in healthy volunteers, and the decrease in these miRs was concordant with the decline in the glomerular filtration rate [70]. Other investigators found decreased levels of miR-15b, which has targets involved in phosphate metabolism, in 30 CKD patients compared with 20 healthy controls. In this study, circulating miR-15b was found to correlate positively with estimated glomerular filtration rate (r = 0.502, p = 0.003) and negatively with phosphate levels (r = −0.516, *p* = 0.004) [71].

One study reviewed existing literature to identify circulating miRs common to CKD, CAD and diabetes mellitus, all diseases that have a high prevalence of vascular calcification. In this analysis, which included 15 studies, seven miRs were found to be common to at least two of the three diseases (miR-21, miR-27, miR-34a, miR-126, miR-146a, miR-155 and miR-210). miR-21 was the only miR that was found to be common among all of the diseases, which is not surprising, as miR-21 targets BMPR2 and is, therefore, likely involved in the calcification process. Interestingly, when miR-21 levels were measured in the serum and calcified atherosclerotic and peripheral arterial disease vessels, they were elevated in the vessels and decreased in serum. This may be explained, in part, by the timing of measurements relative to calcification, as well as the concept that serum levels may have been depleted after the delivery of miR-21 to the vessels [72]. Although the aforementioned studies provide some early insight into the relationship between circulating miRs and vascular calcification, more definitive information will be obtained with the completion of the MAP-Calcification Study (ClinicalTrials.gov: NCT01992848). This study will enroll 100 patients using a case-control study design to identify miRs as putative biomarkers for coronary calcification.

## 7. miRs and Osteoclastogenesis

Another theory of vascular calcification posits that, similar to bone formation, which is a dynamic process that involves simultaneous bone matrix elaboration by osteoblasts and resorption by osteoclasts, the osteoclast-like cells are deficient in either number or activity [14]. The contribution of osteoclastogenesis and osteoclast-like cells to vascular calcification remains somewhat controversial; however, the finding of osteoclast-like cells in calcified areas of vessel indicates that this is likely to be a significant component of the vascular calcification process [39]. The integrated nature of bone formation and resorption has been described in studies performed in Runx2 knockout mice. These mice have a complete lack of normal bone formation that is characterized by an absence of osteoclasts, suggesting that Runx2 regulates the expression of a factor necessary for osteoclast formation [73]. While levels of the osteoclast inhibitory factor, osteoprotegerin, were found to be normal, the levels of the receptor activator of nuclear factor-kappaβ ligand (RANKL), which promotes osteoclast differentiation, were diminished. Using a Runx2 null calvaria-derived cell line (CA120-4), osteoclast differentiation from normal bone marrow cells in co-culture was decreased significantly in the absence of Runx2. Confirmatory studies performed using adenovirus-mediated gene transfer of Runx2 further demonstrated normal osteoclast differentiation from bone marrow cells, indicating that Runx2 promotes osteoclast differentiation by inducing RANKL and inhibiting osteoprotegerin [74].

The link between calcifying vascular SMCs and osteoclast-like cells has also been examined. *In vitro* studies confirmed that once SMC transdifferentiated to osteoblast-like cells and expressed Runx2, Runx2 increased the expression and secretion of RANKL by binding directly to the RANKL promoter. This, in turn, resulted in the migration and differentiation of bone marrow-derived macrophages. These bone marrow-derived macrophages transitioned into multinucleated tartrate-resistant acid phosphatase-positive functional osteoclast-like cells. The obligate role of Runx2 was confirmed by performing similar *in vitro* studies with Runx2 null SMC with diminished RANKL expression [75]. Subsequent *in vivo* studies using a high-fat diet as the stimulus to induce vascular calcification revealed that SMC-Runx2-deficient mice had decreased vessel mineralization, and this finding was accompanied by a decrease in RANKL, as well as limited vascular macrophage infiltration and formation of osteoclast-like cells. Thus, vascular calcification is coupled to the formation of osteoclast-like cells, similar to what is observed for skeletal bone formation [76].

The role of miRs in regulating osteoclastogenesis was defined by examining osteoclast precursors with a global deletion of the miR processing factors, Dgcr8, Dicer or Ago2. The consequence of inhibiting global miR expression was that osteoclast differentiation was prevented owing to a decrease in the osteoclast transcription factors, PU.1, Mitf, fos and Nfatc1 [77]. Studies performed in mice with Dgcr8 deficiency confirmed this phenomenon by demonstrating impaired osteoclast function, which resulted in an increase in bone mass [78].

Although there have been no studies that have directly examined the role of miRs in regulating the differentiation of monocytes/macrophages to osteoclast-like cells in calcifying vessels, it is highly plausible that the same miRs that regulate osteoclast function are operative in vascular calcification (Figure 1). This speculation is supported by the fact that there are several miRs that have been linked to both osteoclastogenesis, as well as cardiovascular disease. In particular, miR-126, which targets RANKL, is decreased in patients with diabetes mellitus and coronary artery disease, who have a high prevalence of vascular calcification [79,80]. Similarly, miR-146a, which is expressed in atherosclerotic arteries, inhibits osteoclastogenesis by limiting the number of tartrate-resistant acid phosphatase positive cells [81,82].

It is notable that miR-34a, which is related to the Runx2-targeting miR-34c, has been identified as a potent inhibitor of osteoclastogenesis. miR-34a knockdown mice had evidence of increased bone resorption and decreased bone mass, while transgenic miR-34 mice exhibited the opposite effect. Studies found that miR-34 targeted the pro-osteoclastogenic factor transforming growth factor-β-induced factor 2 (Tgif2). *In vivo* studies identified an important regulatory role for miR-34 by demonstrating that it was involved in osteoporosis and cancer-related bone metastasis [83]. Similarly, miR-29b, which mediates Wnt signaling in calcifying SMC is decreased progressively in human osteoclasts during osteoclastogenesis. Overexpression of miR-29 confirmed that it regulates osteoclastogenesis by targeting Nfatc1, cathepsin K, matrix metalloproteinase-9, tartrate resistant acid phosphatase and RANK expression, leading to a decrease in bone resorption activity and extracellular matrix degradation [84]. In contrast, studies performed in mice showed that miR-29b is upregulated in bone marrow-derived cells during osteoclastogenesis, suggesting that osteoclast-regulating miRs may have different functions in different cell types [85]. 

Other miRs have also been implicated in the regulation of osteoclast function. In postmenopausal women with osteoporosis and increased osteoclast activity, miR-503 was downregulated in CD14+ peripheral blood mononuclear cells. *In vitro* analysis confirmed that miR-503 targeted the osteoclast receptor RANK and was regulated by 17 β-estradiol [57]. Thus, it is likely that miR-503 is upregulated in vascular calcification, where osteoclastogenesis is deficient. Similarly, miR-148a was identified as a highly upregulated miR in peripheral blood mononuclear cells and found to target V-maf musculoaponeurotic fibrosarcoma oncogene homolog B (Mafb), a negative regulator of osteoclastogenesis [86]. miR-125a was also found to be downregulated during osteoclastogenesis in peripheral blood mononuclear cells. Downregulation of this miR led to upregulation of its direct target tumor necrosis factor receptor associated factor 6, which is essential for normal osteoclast function. In addition, the osteoclast transcription factor Nfatc1 was shown to bind to the miR-125a promoter and inhibit its transcription, thereby identifying a feedback loop [87]. Taken together, there is compelling evidence to indicate that miRs govern osteoclast maturation and function and that these findings can be extrapolated to vascular calcification.

## 8. Conclusions

Vascular calcification is a severe vascular end pathophenotype that is associated with vascular dysfunction and is not reversible. Vascular medial calcification, in particular, may occur in the absence of atherosclerosis and is prevalent in diabetes mellitus, CKD and with aging. The processes that govern vascular medial calcification are incompletely understood, but include transdifferentiation of SMC to osteoblast-like cells, release of circulating matrix vesicles containing calcium phosphate that serve as nucleation sites for calcification and a decreased number or activity of osteoclast-like cells that resorb bone matrix. Each of these pathophysiological processes involves genetic reprogramming that is regulated by miRs. Changes in miR expression have been shown to mediate SMC and monocyte acquisition of an osteoblast- or osteoclast-like phenotype, respectively. Moreover, the finding of several unique miRs, as well as several common miRs is indicative of redundancy in the system. It is likely that these miRs are able to regulate phenotype shifts via distinctive miR programs with temporal and cell-specific signatures that initiate SMC calcification. Future studies that identify these programs will identify master regulatory miRs that are amenable to therapeutic intervention.

## Abbreviations

SQ: subcutaneous
MAP-Calcification Study: MicroRNAs as Potential Biomarkers for Coronary Artery Calcification Study
